# Simultaneous fatal poisoning of two victims with 4F-MDMB-BINACA and ethanol

**DOI:** 10.1007/s11419-022-00632-y

**Published:** 2022-06-28

**Authors:** Gábor Simon, Dénes Tóth, Veronika Heckmann, Mátyás Mayer, Mónika Kuzma

**Affiliations:** Department of Forensic Medicine, Medical School, University of Pécs, Pécs, Hungary

**Keywords:** Synthetic cannabinoid receptor agonist, 4F-MDMB-BINACA, Ethanol, Overdose, Poisoning

## Abstract

**Purpose:**

Methyl-2-(1-(4-fluorobutyl)-1H-indazole-3-carboxamido)-3,3-dimethylbutanoate (4F-MDMB-BINACA) is a newly emerging synthetic cannabinoid receptor agonists (SCRA) first described in 2018 in both Europe and the United States. Two fatal cases are reported caused by simultaneous consumption of 4F-MDMB-BINACA and ethanol.

**Methods:**

The victims were brothers who were both found deceased after consuming 4F-MDMB-BINACA and ethanol. Post-mortem toxicological analyses of blood and urine were carried out by supercritical fluid chromatography tandem mass spectrometry (SFC–MS/MS) and headspace gas chromatography with flame ionization detection (HS-GC–FID).

**Results:**

The concentration of 4F-MDMB-BINACA in the postmortem blood was 2.50 and 2.34 ng/mL, and blood alcohol concentration was 2.11 and 2.49 g/L, respectively.

**Conclusion:**

According to the reported cases and reviews of the scientific literature, concurrent ethanol consumption should amplify the toxicity of SCRAs. The threshold SCRA concentration for fatal overdose can be estimated ng/mL level (0.37–4.1 ng/mL according to the reported cases) in cases in which 1.5–2.5 g/L of ethanol is present in the blood.

## Introduction

Synthetic cannabinoid receptor agonists (SCRAs) gained popularity in the late 2000s [1]. The limited information to date regarding their pharmacology and toxicology and the increasing diversity in their structures raises public health concerns [2]. Severe adverse effects including psychosis, arrhythmia, hemorrhagic stroke, acute kidney injury, and acute pancreatitis are frequently associated with SCRA use [3–5]. Due to the unknown toxicity of newly emerging SCRAs, forensic assessments of cases involving these substances are challenging. Direct drug toxicity is the most common diagnosis for SCRA-related fatalities [3] and can be a contributing factor of death in the presence of pre-existing disorders, such as cardiovascular disorders (e.g., fibrosis and hypertrophy) and pulmonary disorders (e.g., pneumonia) [6, 7]. SCRAs are rarely consumed in isolation; rather, they are often used in combination with other psychoactive substances, especially other SCRAs or alcohol [8, 9]. Data concerning the combined effects of SCRAs and other substances are highly limited, which renders forensic evaluation of possible overdose cases difficult [10]. Given that only a few well-documented studies are available in the literature, individual case reports are critical sources for comprehensive and accurate assessments of SCRA-related fatalities [7].

**Fig. 1:**
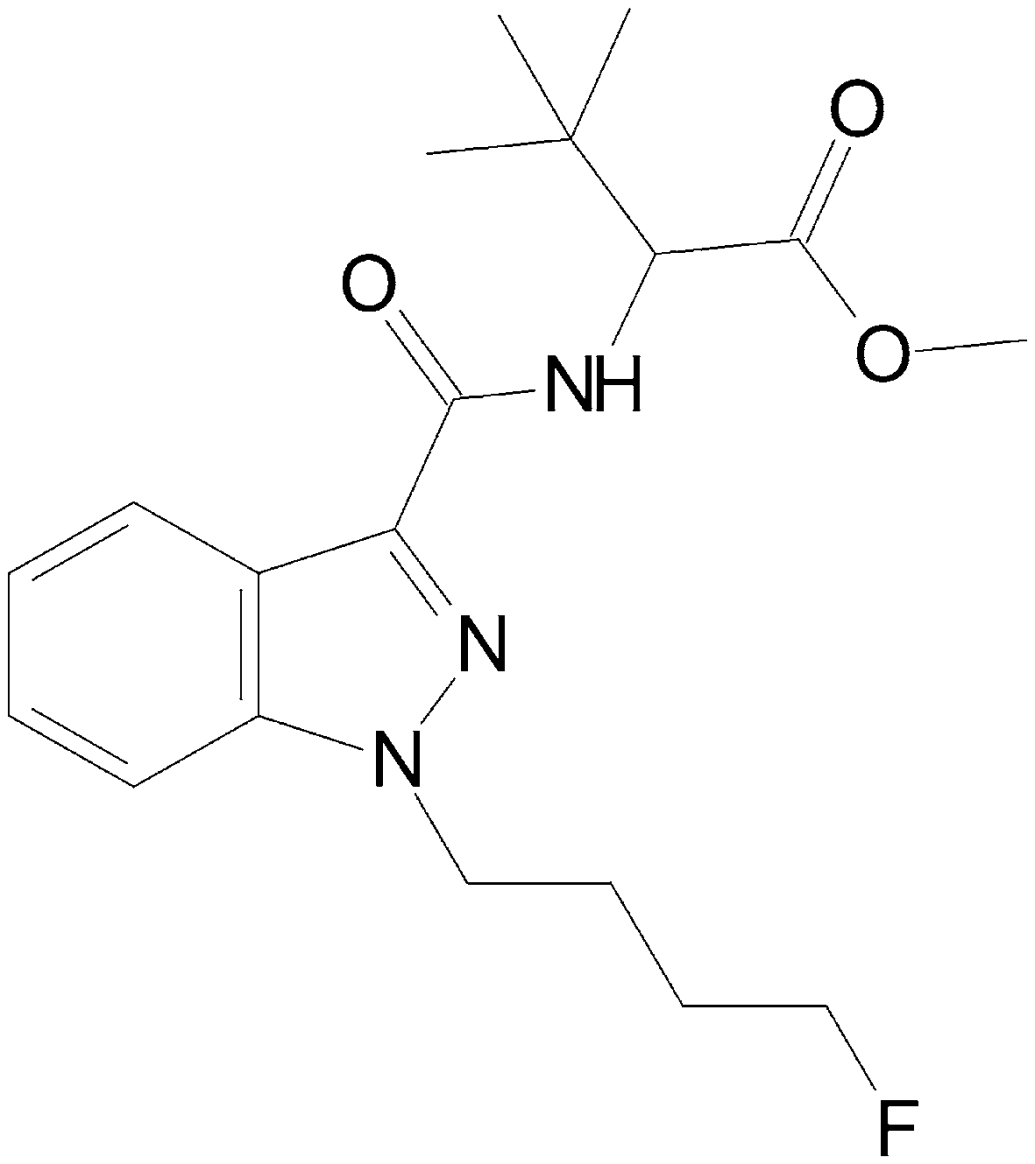
Chemical structure of 4F-MDMB-BINACA

4F-MDMB-BINACA — also known as 4F-MDMB-BUTINACA or 4F-ADB, IUPAC name: methyl-2-(1-(4-fluorobutyl)-1H-indazole-3-carboxamido)-3,3-dimethylbutanoate (Fig. 1) — is a fluorinated, cannabimimetic indazole carboxamide derivative that was first described in 2018 [9]. It is usually available as a powder, liquid (vapor fluid), or herbal plant mixture. 4F-MDMB-BINACA was commonly found in conjunction with methyl-(*2*S)-2-[[1-(5-fluoropentyl)indole-3-carbonyl]amino]-3,3-dimethylbutanoate (5F-MDMB-PICA) [9, 11], and in 2020, it was amended into Schedule II of the Convention on Psychotropic Substances of 1971 [12].

4F-MDMB-BINACA is potent cannabinoid receptor type 1 (CB1) and cannabinoid receptor type 2 (CB2) agonist [13, 14]. Binding of SCRAs to the CB1 stimulates pertussis toxin-sensitive G proteins (Gi/Go) resulting in the inhibition of adenylyl cyclase, a decreased opening of N-type Ca^2+^ channels and the activation of G protein-gated inward rectifier (GIRK) channels [15]. Phytocannabinoids, such as CBD (cannabidiol) and THC (tetrahydrocannabinol), have affinity for other receptors also, but it is not known, whether SCRAs are ligands of these [16]. The half-maximal effective concentration (EC_50_) of 4F-MDMB-BINACA is 5.69 nM (2.76–11.0 nM) on CB1, and 0.69 nM (0.30–1.56 nM) on CB2, in vitro half-life (t_1/2_) is 10.27 min [14]. Reliable data about dosage are unavailable [9].

### Case report

**Fig. 2:**
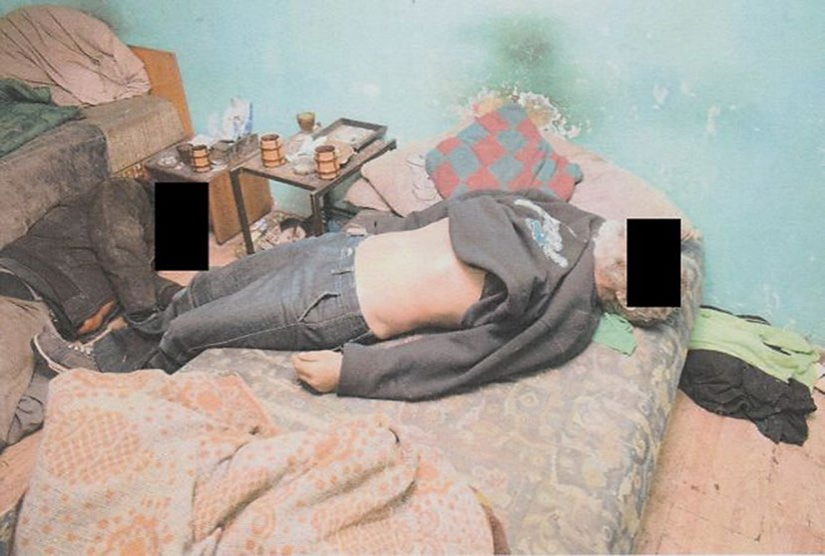
Position of the victims. Victim A (left), Victim B (right)

A 45-year-old male (Victim A) and a 50-year-old male (Victim B) were found deceased by their brother (Witness A) around 11:00 p.m. They had begun drinking around 1:00 p.m., consuming white wine purchased from their local shop, red wine bought from a friend from the same village, and homemade hard liquor. Victim B also brought “something resembling a drug” (unrecognizable by Witness A) from his cousin (Witness B) in a cigarette box and mixed this substance with their tobacco. Witness A did not consume this tobacco and fell asleep shortly thereafter. When he awoke a few hours later, he discovered both victims deceased. Victim A was found lying on the ground and Victim B lying on the bed (Fig. 2). According to Witness A, the victims had not previously used drugs, but they reportedly consumed ethanol daily (a little liters of beer or wine per day). Medical history was negative for Victim A, whereas Victim B had hypertension, although witnesses attest that he failed to take his medication regularly. According to Witness B, Victim B found the cigarette box when they were hauling used furniture acquired during a disposal. The cigarette box contained light-green-colored marjoram-like material wrapped in aluminum foil.

During the death scene examination, multiple cigarette butts without filters were found in an ashtray; also found were alcohol bottles, an unopened box of nebivolol-containing drug, and 18 g of unrecognizable herbal residue in a cigarette box. The rectal temperature of Victim A was 28.4 °C (recorded at 2:30 a.m. the following day). The large, dark-red-colored hypostatic patches did not fade away with pressure, and rigor mortis was present in all extremities. The rectal temperature of Victim B was 31.4 °C, the dark-red-colored hypostatic patches faded away slightly with pressure, and rigor mortis was present in all extremities, reappearing shortly after moving the limbs. The room temperature was 9.2 °C.

### Materials and methods

#### Autopsy and histological examinations

Forensic autopsy of both victims was performed four days after the time of death following the Recommendation No.R (99)3 of the Council of Europe [17] on medico-legal autopsies. Toxicological samples were collected from the femoral vein (whole blood) and bladder (urine). Histological samples were collected from the brain (posterior limb of the internal capsule with adjacent thalamus, rostral pons, cerebellum including the dentate nucleus, hippocampus, dorsal frontal inter-arterial border zone), lung, heart, kidney, and liver. Histological samples were collected from the following areas of the heart: sinoatrial node, Koch’s triangle (AV node), right ventricle, septum, and left ventricle. All samples were fixed with 9% buffered formalin and stained with hematoxylin and eosin (HE). Immuno-histological staining for complement factor C9 (cat. no. ABS 004–22-02, Thermo Fisher Scientific, Rockford, IL, USA) was applied on all heart samples.

#### Toxicological analyses

Toxicological analyses were carried out by supercritical fluid chromatography tandem mass spectrometry (SFC–MS/MS) (Waters^®^ ACQUITY UPC^2^ supercritical fluid chromatograph coupled with Xevo TQ-S triple quadrupole mass spectrometer, Milford, MA, USA), and headspace gas chromatography with flame ionization detection (HS-GC–FID) (Agilent Technologies G1888 headspace with 7890A gas chromatograph system, Santa Clara, CA, USA). SFC-MS/MS was used to identify and quantify 295 compounds, including drugs (e.g., antihypertensive drugs, anxiolytics, antipsychotics, antidepressants, antiepileptics, general and local anesthetics, NSAIDs, opioids, anticoagulants), narcotics, and novel psychoactive substances (e.g., SCRAs, phenethylamines, tryptamine derivatives). Metabolites of SCRAs are not analyzed routinely in our laboratory. Prior to the extraction, nine isotope-labelled internal standards were added to the samples, namely amphetamine-D6, 4-methylmethcathinone-D3, delta-9-Tetrahydrocannabinol-D3, 11-nor-9-carboxy-Tetrahydrocannabinol-D3, N-[(1S)-1-(aminocarbonyl)-2-methylpropyl]-1-[(4-fluorophenyl)methyl]-1H-indazole-3-carboxamide-D4 (AB-FUBINACA-D4), carbamazepine-D10, citalopram-D6, alprazolam-D5, and clonazepam-D4. HS-GC-FID method was applied for the determination of alcohols (methanol, ethanol, 1-propanol, 2-propanol, *n*-butanol) and other volatiles (e.g., acetone, toluene, ethyl acetate). *Tert*-butanol was used as an internal standard.

#### SFC–MS/MS conditions

Measurements were performed by an ACQUITY UPC^2^ supercritical fluid chromatography system (Waters) coupled with a Xevo TQ-S Triple Quadrupole Mass Spectrometer (Waters). Data were recorded by MassLynx software.

Separation of compounds was performed on a 2.1 mm×100 mm, 1.7 μm particle size ACQUITY Torus™ DIOL analytical column (Waters) with guard cartridge. Chromatography was performed at 45 °C and the injected volume was 0.5 μL. The flow rate of the mobile phase was 0.6 mL/min. The mobile phase consisted of a mixture of carbon dioxide (A) and 10 mM ammonium hydroxide and 12 mM formic acid in methanol/water (97.2/2.8, v/v) (B). The following gradient profile was used: 97.5% A at 0 min and 37.5% A at 10 min. A pre-equilibration period lasting 2.5 min was applied before each injection. Constant 175 bar back pressure was used to maintain the supercritical state. To sustain, a suitable electrospray methanol was used with a flow rate of 60 μL/min, this makeup solvent was delivered by a Waters 515 HPLC Pump. The MS measurement was performed in positive ion mode (except for some acidic compounds such as barbiturates). The ESI source was operated with a spray voltage of 3 kV in both positive and negative ion modes. Cone voltage was 30 V. The source was set at 150 °C. Both desolvation and cone gases were nitrogen delivered at 300 and 150 L/min, respectively. Desolvation gas was tempered at 300 °C. The collision gas was argon with a flow rate of 0.13 mL/min. MS/MS experiments were performed in MRM (multiple reaction monitoring) mode with an isolation window of 0.4 *m*/z. Peak detection and quantification were achieved using TargetLynx XS software (Waters). The observed ions (mass in *m/z*) were accepted and quantified if the following conditions were met: appropriate MS1 mass, appropriate retention time, appropriate MS2 mass, appropriate fragmentation pattern and internal standard correction.

#### Sample preparation – SALLE (salting out assisted liquid–liquid extraction)

One hundred and twenty μL of internal standard solution (125 mM formic acid/acetonitrile) was added to 90 μL of the sample. After vortex-mixing, the mixture was allowed to stand at room temperature for 5 min. In the next step, ammonium formate as salting agent was added to the mixture and incubated in a thermomixer (20 °C, 1200 rpm) for 15 min. After the incubation, mixture was centrifuged (18,000 x g, 20 °C) for 5 min and 0.5 μL of the supernatant was directly injected to the chromatographic system.

### Autopsy results

#### Victim A

The body of victim was 170 cm in height, medium in build, and well nourished. The skin was pale, and dark-red-colored hypostatic patches were observed on the back, on the left side of the chest, and on the neck. Rigor mortis was present in the upper and lower extremities. Petechial bleedings were not observed in either the skin or conjunctiva. Abrasions showing healing signs were seen on the forehead, left forearm, and right shin. During the internal examination, slight brain edema; dark-reddish and fluid blood; dilatation of atriums and ventricles; minimal lung edema; congestion of lung, liver, spleen, and kidney; and mild atherosclerosis were recorded. The organ weights were as follow: brain 1230 g, heart 370 g, spleen 105 g, liver 1450 g, kidney 291 g. Histopathological examinations revealed acute congestion in all organs. Additionally, mild liver steatosis, patchy pulmonary edema, and mild sub-endocardial fibrosis were detected. Immuno-histochemical staining with the early hypoxia marker complement component C9 was negative in all heart samples. Toxicological analyses confirmed the presence of 4F-MDMB-BINACA, theophylline, caffeine, and ethanol in the blood (Table 1).

**Table 1:** Summary of toxicological findings

	Victim A		Victim B	
Sample	Whole blood	Urine	Whole blood	Urine
4F-MDMB-BINACA	2.50 ng/mL	ND	2.34 ng/mL	ND
Theophylline	938 ng/mL	1177 ng/mL	268 ng/mL	960 ng/mL
Caffeine	5760 ng/mL	> 3500 ng/mL	3140 ng/mL	3160 ng/mL
Paracetamol	ND	11 ng/mL	9 ng/mL	384 ng/mL
Nebivolol	ND	ND	ND	1920 ng/mL
Ethanol	2.11 g/L	3.12 g/L	2.49 g/L	3.33 g/L

*LOQ* (limit of detection) was 0.02 ng/mL for 4F-MDMB-BINACA, 5 ng/mL for paracetamol, 5 ng/mL for nebivolol, and 0.05 g/L for ethanol (in whole blood and in urine, respectively)

*ND* not detected

#### Victim B

The body of victim was 172 cm in height, medium in build, and well nourished. The skin was pale, and dark-red-colored hypostatic patches were observed on the back and on both sides of the trunk. Rigor mortis was present in the upper and lower extremities. Neither petechial bleedings nor injuries were detected during the external examination. During the internal examination were found: slight brain edema; dark reddish and fluid blood; mild atherosclerosis causing no significant occlusion; dilatation of atriums and ventricles; acute bronchitis; lung oedema; and congestion of lung, liver, spleen, and kidney. The organ weights were as follows: brain 1444 g, heart 312 g, spleen 190 g, liver 1687 g, kidney 277 g. Microscopically, internal congestion, mild liver steatosis, and patchy pulmonary edema were detected. Mild sub-endocardial and perivascular fibrosis were present in the heart. Complement component C9 immunohistochemistry was negative in all heart samples. Toxicological analyses revealed the presence of 4F-MDMB-BINACA, theophylline, caffeine, paracetamol, and ethanol in the blood (Table 1).

## Discussion

Reported fatalities related to SCRAs suggest that their effects are unpredictable, and no threshold for a lethal dose can be determined. Concentrations of SCRAs in postmortem cases cover a wide range [18]; however, some reports of survival have also been published—even at relatively high blood SCRA concentrations [19, 20]. In SCRA-related cases in which the deceased suffered from heart disease, the SCRA concentration in the postmortem blood was less than 1 ng/mL [18]. Although the lethal dose of 4F-MDMB-BINACA is unknown, its concentration in postmortem blood samples was found to range between 0.10 and 2.90 ng/mL [21].

This report presents two fatal cases associated with simultaneous 4F-MDMB-BINACA and ethanol abuse. Our findings revealed that both victims consumed large amounts of alcohol preceding their deaths (blood alcohol concentrations (BAC) were 2.11 and 2.49 g/L, respectively). Concentrations of 4F-MDMB-BINACA in the postmortem blood samples were 2.50 and 2.34 ng/mL, which are in line with published data. The fact that 4F-MDMB-BINACA was not detected in postmortem urine samples is partly explained by the high rate of hepatic metabolism of SCRAs [11, 14, 22], but also suggests that the victims consumed 4F-MDMB-BINACA shortly before their deaths.

SCRAs are often consumed together with ethanol, which is detected nearly half of the recorded cases of SCRA-related deaths [8, 23]. Several case reports describe that the presence of a little ng/mL (0.37–4.1) of SCRAs and a high—but not lethal—concentration of ethanol (1.45–2.7 g/L) directly and exclusively contributed to the death of the victim [24–27] (Table 2). No case report to date describes an SCRA level  >  5 ng/mL concurrent with a higher ethanol concentration (>  1.5 g/L) [18].

**Table 2:** Reported cases of simultaneous consumption of SCRAs and ethanol

Age (y)/Sex	SCRA and concentration	BAC	Study
33/F	5F-MDMB-PICA 1.7 ng/mL	2.2 g/L	Kleis et al. [21]
25/M	5F-PB-22 0.37 ng/mL	2.5 g/L	Angerer et al. [22]
28/M	AB-CHMINACA 4.1 ng/mL	1.45 g/L	Angerer et al. [22]
30/M	AB-CHMINACA 1.5 ng/mL	1.8 g/L	Gieron et al. [23]
51/M	Cumyl-PEGACLONE 0.73 ng/mL	2.7 g/L	Tiemensma et al. [24]
50/M	Cumyl-PEGACLONE 3.0 ng/mL	2.4 g/L	Tiemensma et al. [24]
33/M	Cumyl-PEGACLONE 2.0 ng/mL	2.4 g/L	Tiemensma et al. [24]

Very limited data are available in the scientific literature about the possible effects of the combined consumption of SCRAs and ethanol. Funada et al. described that ethanol-induced motor impairments were enhanced by synthetic cannabinoids in mice [28], although no human studies exist on this topic to date. Given that THC and ethanol act on the same receptors, data on their simultaneous use may yield important insights in this regard. Studies have found no unequivocal synergistic effect between THC and ethanol at low or moderate ethanol doses [29, 30], but no data on high doses of ethanol are available. Both ethanol and SCRAs can cause respiratory depression [31–33], which may underlie the dangers of their combined use.

Endocannabinoid system plays an important role in the regulation of respiration by modulating the respiratory rate, and this mechanism is dependent on CB1 [34]. The CB1 is present in several nuclei belonging to these respiratory center [35]. Wiese et al. had shown, that a selective CB1 agonist SCRA induces respiratory depression, while selective CB2 agonist SCRA does not have a significant effect on respiration [36]. Their study suggests, that respiratory depression effect of SCRA is probably caused by CB1 activation in the respiratory center, probably the isolated CB1 activation in the preBötzinger complex of the brain stem [37].

Van Rafelghem et al. published the first detailed clinico-pathological description of lethal intoxication by 4F-MDMB-BINACA following extensive vaping. The victim died due to severe necrotizing pancreatitis and acute kidney injury evolving into multi-organ failure 11 days after hospital admission [4]. Van Rafelghem et al. hypothesized that acute pancreatitis and acute kidney injury were induced by the tissue toxicity of 4F-MDMB-BINACA via CB1 activation and by the toxic effect of terminal fluorination [4]. It is known that terminal fluorination of SCRAs increases the potency of SCRAs at CB1 [37]. In addition, excessive fluoride intake has been linked with damage to soft tissues [38], including nephrotoxicity and acute kidney injury [39, 40].

The fact that similar 4F-MDMB-BINACA and ethanol concentrations were detected in the postmortem blood samples of both victims suggests that both substances played a role in the fatal outcome. The victims did not have any significant diseases that could have contributed to the outcome. These results indicate that the simultaneous intoxication of SCRA and ethanol directly and exclusively caused the death of the two victims. The direct cause of death was determined as respiratory depression by the combined use of 4F-MDMB-BINACA and ethanol.

## Conclusion

Ethanol is the most frequently co-detected substance in fatal cases involving SCRAs. The reported cases and reviews of the scientific literature suggest a possible synergistic effect between SCRAs and ethanol, because their combined use clearly increases their toxicity. The threshold for fatal overdose of combined use of SCRAs and ethanol can be estimated as a little ng/mL (0.37–4.1 ng/mL according to the reported cases) of SCRA and 1.5–2.5 g/L of ethanol.
